# The role of new inflammatory indices in the prediction of endoscopic and histological activity in inflammatory bowel disease patients

**DOI:** 10.1097/MEG.0000000000002842

**Published:** 2024-09-12

**Authors:** Nicola Cesaro, Marco Valvano, Sabrina Monaco, Gianpiero Stefanelli, Stefano Fabiani, Filippo Vernia, Stefano Necozione, Angelo Viscido, Giovanni Latella

**Affiliations:** a Division of Gastroenterology, Hepatology, and Nutrition, Department of Life, Health and Environmental Sciences, University of L’Aquila, Piazzale Salvatore Tommasi, L’Aquila, Italy; b Division of Gastroenterology, Galliera Hospital, Genoa, Italy; c Epidemiology Unit, Department of Life, Health and Environmental Sciences, University of L’Aquila, italy.

**Keywords:** Crohn’s disease, C-reactive protein albumin ratio, inflammatory bowel disease, inflammatory index, systemic inflammation response index, ulcerative colitis

## Abstract

**Background and aim:**

Inflammatory indices are promising indicators that can be used to evaluate inflammation in inflammatory bowel diseases (IBDs). The present study aimed to investigate the test accuracy of several inflammatory indices to identify endoscopic, and histological activity in a cohort of IBD patients.

**Study:**

All IBD patients who underwent colonoscopy and blood examination (within 4 weeks and without therapeutic change) were included. For these patients, 10 different inflammatory biomarkers were collected. Our primary outcome was the assessment of accuracy [evaluated with a receiver operating characteristics (ROC) analysis] of each inflammatory biomarker and indices. Furthermore, we tried to establish the optimal cutoff to identify patients with endoscopic and histologic activity among the inflammatory biomarkers and indices with higher performance.

**Results:**

Regarding endoscopic activity, at the ROC analysis, the systemic inflammation response index (SIRI) showed the best accuracy [area under the curve (AUC), 0.627; confidence interval (CI), 0.552–0.698]. Whereas the ROC analysis showed a suboptimal AUC for the neutrophil-to-lymphocytes ratio (NLR) and platelets-to-lymphocytes ratio; (AUC, 0.620; CI, 0.545–0.691 and AUC, 0.607; CI, 0.532–0.679, respectively). Concerning histological activity, the C-reactive protein albumin ratio (CAR) presented a higher accuracy among the calculated inflammatory biomarkers (AUC, 0.682; CI, 0.569–0.781) while SIRI and NLR presented a subdued diagnostic performance.

**Conclusion:**

SIRI and CAR presented the best test accuracy in an IBD outpatient setting to identify endoscopic and histological activity. However, the test accuracy of all the evaluated Inflammatory indices appeared suboptimal. Fecal calprotectin has still the highest accuracy in predicting endoscopic and histological activity in patients with IBD.

## Introduction

Inflammatory bowel diseases (IBDs) are chronic relapsing inflammatory disorders of the gastrointestinal (GI) tract characterized by relapsing periods alternate with quiescent periods of clinical activity [1]. IBDs include ulcerative colitis (UC), which exclusively affects the rectum and colon, and Crohn’s disease (CD), which may affect any GI tract, but predominantly the distal ileum and right colon [2,3]. In the last decades, the prevalence of IBD has increased, probably linked to urbanization, dietary modifications, antimicrobial use, and factors affecting host–gut microbiome homeostasis [4].

IBDs require lifelong medications because of the progressive structural and functional alterations of the involved GI tracts [5]. Over the years, broad-spectrum and disease-specific drugs have been developed, obtaining major improvements in clinical outcomes and patients’ quality of life [6,7]. Despite these therapeutic advances, a significant proportion of patients do not respond to therapy or lose response [8]. The mechanisms underlying the loss of response are not completely known. The possibility of predicting the response to the chosen treatment would allow patients, with possible refractoriness, to receive the best therapeutic option [8].

The identification of numerous potential biomarkers has improved the management of IBDs. Biomarkers are useful for diagnosis, assessment of disease activity, and prediction of response to therapy [9,10].

European Crohn´s and Colitis Organisation-European Society of Gastrointestinal and Abdominal Radiology guidelines indicate fecal calprotectin (FC) and C-reactive protein (CRP) as useful biomarkers in the management of IBD [8].

The utility of FC monitoring in patients with quiescent disease was evaluated in a recently published systematic review, in particular, two elevated values consecutively of FC are associated with disease relapse (53–83%) in the subsequent 2–3 months [11]. In a recent meta-analysis, FC in diagnosing active disease, both in CD and UC, showed a pooled sensitivity of 85%, specificity of 75%, and area under the curve (AUC) of 0.88. The subgroup analysis revealed that FC performed better in UC than in CD in assessing endoscopic activity (pooled sensitivity 87.3% vs. 82.4%, specificity 77.1% vs. 72.1%, and AUC 0.91 vs. 0.84) [12]. Currently, there is no agreement on the ideal cutoff point for FC for disease monitoring. In clinical trials where the response to a new treatment is monitored, a low cutoff point (e.g. 100 μg/g) is frequently used, while in real-life studies, a higher cutoff point is utilized (e.g. 250 μg/g) [13–15].

Serum CRP is an acute-phase reactant that has been considered for many years as a general indicator of inflammation. The CRP is a noninvasive biomarker nonspecific for gut inflammation. It can be normal in up to 30% of patients despite an active disease with a suboptimal correlation between activity index and CRP levels in both CD and UC [16]. In a meta-analysis that compared its diagnostic accuracy, CRP correlated with endoscopy in patients with symptomatic IBD; a CRP concentration of ≥5 mg/L appeared to have a high specificity for detecting endoscopic disease activity with an AUC of 0.72 (95% CI, 0.68–0.76). However, the sensitivity was very poor, and a negative CRP value does not exclude the presence of a relapsing disease [17]. Repeated CRP measurements in early postoperative recurrence of CD or in follow-up of small bowel CD are less useful against repeated FC measurements [18,19].

Several noninvasive, easily accessible, cost-effective, and feasible inflammatory indices have been reported including systemic inflammation response index (SIRI), neutrophil-to-lymphocytes ratio (NLR), platelets-to-lymphocytes ratio (PLR), lymphocytes-to-monocytes ratio (LMR), eosinophil-to-lymphocytes ratio (ELR), eosinophil and neutrophil-to-lymphocytes ratio (ENLR), C-reactive protein albumin ratio (CAR), systemic immune inflammation index (SII), monocytes-to-lymphocytes ratio (MLR), and aggregate index of systemic inflammation (AISI).

These indices are considered useful indicators of the local and systemic inflammatory response, and their prognostic value has been evaluated in several diseases.

In patients with different kinds of cancer (hepatic, pancreatic, gastric, and renal tumors), high levels of some of these indices (SIRI, NRL, PLR, LMR, ELR, ENLR, and CAR) before treatment were associated with poor prognosis [20–24].

SII and SIRI were effective in predicting the presence of atrial fibrillation in patients with ischemic stroke [25].

Some of these indices such as PLR, MLR, NLR, neutrophil/lymphocyte platelet ratio, AISI, SIRI, and SII have been reported to be effective in predicting the severity and mortality in patients with COVID-19 and the severity of rheumatoid arthritis [26–29].

SIRI, SII, CAR, NRL, and PLR have been shown to have prognostic value in predicting clinical and endoscopic activity and severity in patients with UC and CD. The high values of these indexes are associated with active IBD and may help clinicians make correct decisions in the management of these patients [30–34].

The present study aimed to investigate the test accuracy of inflammatory biomarkers and indices to identify endoscopic, and histological activity in a cohort of adult IBD patients.

## Materials and methods

### Study design and population

We retrospectively collected the clinical data of all IBD patients followed to the Gastroenterology Unit of ‘San Salvatore’ Hospital of L’Aquila (Italy), who underwent colonoscopy from January 2020 to January 2023 with a concomitant serological evaluation within 4 weeks were included.

All clinical investigations were conducted according to the principles laid down in the Declaration of Helsinki and reported according to the Strengthening the Reporting of Observational Studies in Epidemiology Statement guideline [35]. The study was approved by the Internal Review Board of the University of L’Aquila, Italy (protocol number: 12/2023, March 2023). All patients gave their consent to participate in the current study and data processing.

### Inclusion and exclusion criteria

IBD patients who underwent colonoscopy with a concomitant blood examination (within 4 weeks and without therapeutic change) were included. All IBD patients were considered eligible, irrespective of disease activity (remission or active disease) and administered therapies (biological or conventional).

#### Inclusion criteria

The inclusion criteria were: (1) Definite diagnosis of IBD [8]; and (2) colonoscopy with a concomitant blood examination (within 4 weeks and without therapeutic change).

#### Exclusion criteria

The exclusion criteria were: (1) endoscopic examination not available or not present; (2) biochemical examination not present or dated more than 4 weeks after or before the endoscopic examination; (3) therapeutic change (switch, dose escalation, or addition of a new drug in therapy between the endoscopic examination and the blood chemistry tests); (4) the patient who undergoes surgery between the endoscopic examination and the blood chemistry tests; and (5) previous hematological disease.

### Data collection

From all the patients included, the following data were collected: Demographic data (sex, age), type of IBD (UC and CD), disease duration, smoking habit, comorbidity, type of therapy, steroid use, surgery history, clinical score: ‘Harvey-Bradshaw Index’ and Mayo endoscopic score (MES) for CD and UC, respectively, endoscopic activity: MES and simple endoscopic score (SES-CD) for UC and CD, respectively, histological activity, biochemistry (hemoglobin, platelet count, neutrophil, lymphocyte, eosinophil, monocytes, albumin), c-reactive protein (CPR), FC, time between endoscopic and biochemical examination.

According to the MES and the SES-CD, the included patients were categorized as active or inactive group.

MES ≥ 1 or SES-CD ≥ 3 identified endoscopic activity [36].

In all IBD patients who underwent histological examination, at least two biopsies were taken from all colon tracts (left colon, transverse colon, and descending colon) and rectum. In CD patients two more biopsies were taken from the ileum. The histological activity was defined as the presence of inflammatory cell infiltrate in at least one of the histological samples [37].

### Outcome of interest

Using the biochemical parameters collected, the following inflammatory indices were calculated: SIRI, NLR, PLR, LMR, ELR, ENLR, CAR, SII, MLR, and AISI. The formula to calculate the inflammatory indices was reported in Supplementary Table 1, Supplemental digital content 1, http://links.lww.com/EJGH/B60. Our primary outcome was the assessment of accuracy [evaluated with a receiver operating characteristic (ROC) analysis] of each inflammatory biomarker and index. Furthermore, we tried to establish the optimal cutoff to identify patients with endoscopic and histologic activity among the inflammatory biomarkers and indices with higher performance.

### Statistical analysis

For all statistical analyses MedCalc software (version 19.6 MedCalc Ltd., Ostend, Belgium) was used.

The Shapiro-Wilk test was performed to assess the normality of the distribution of collected data. Thus, the data were summarized using absolute and relative frequencies for categorical variables and median with range for the continuous variables.

A Wilcoxon two-sample test was used to compare continuous variables among endoscopic and histologic active and inactive groups.

The AUC and the test accuracy were calculated for each inflammatory marker. A ROC analysis was performed to assess the accuracy of the inflammatory markers. To assess the optimal cutoff among the inflammatory markers with higher performance, Youden’s index test was performed.

The statistical significance level was set at α = 0.05 for all inferential analyses.

## Results

### Included population and baseline characteristics

The baseline demographic and clinical characteristics of the 181 IBD-included patients (66 CD and 115 UC) were reported in Table 1. The median of the disease duration among all the included patients was 9 years (1–76). Concerning CD patients, most of them had an ileal (40%, 28/69) or ileocolic (40%, 26/69) involvement. Among the UC patients, 60% (68/112) had extensive disease. In 157 out of 181 included patients, a complete biopsy sampling was performed; 57% of included patients were on biological therapy, and 34% were under steroids; 45% (80/175) had active disease (according to Partial Mayo Score score for UC and Harvey-Bradshaw Index score for CD), and 17% of patients had a history of previous surgery.

**Table 1.** Baseline demographic and clinical characteristics of the IBD patients

Variable	N/Median (range)	%
Age	49 (16–86)	
Sex		
Male	96/181	53
Female	85/181	47
Type disease		
UC	115/181	63
CD	66/181	37
Disease pattern^a^ (CD)		
A1	6/66	9
A2	40/66	61
A3	20/66	30
L1	28/66	42
L2	12/66	18
L3	26/66	40
L4	0/66	0
B1	28/66	42
B2	28/66	42
B3	10/66	16
Extent of disease (UC)		
E1	15/115	13
E2	32/115	28
E3	68/115	59
Disease duration (years)	9 (1–76)	
Smokers		
Yes	39/167	23
No	128/167	77
Comorbidity		
0	125/181	69
<2	51/181	28
>2	5/181	3
Surgery		
No	150/181	83
Yes	31/181	17
Therapy		
No therapy	20/181	12
Conventional	57/181	31
Biologics	104/181	57
Steroids		
Yes	62/181	34
No	119/181	66
Clinical Score		
PMS	2 (0–8)	
HBI	2 (0–14)	
Endoscopic Score		
MES	2 (0–3)	
SES-CD	2 (0–25)	
Rutgeerts	2 (0–4)	
Histologic activity		
Inactive	38/157	24
Active	119/157	76

A, age at diagnosis (A1: <17 years, A2 : 17–40 years, A3: >40 years); B, Behavior; CD, Crohn’s disease; E, extension; HBI, Harvey-Bradshaw Index; L, Localization; MES, Mayo Endoscopic Score; PMS, Partial Mayo Score; SES-CD, simple endoscopic score; UC, Ulcerative colitis.

a Montreal classification.

### Endoscopic and histologic activity

Concerning endoscopic activity, two groups have been identified. Out of 181 patients, 36 (20%) were in endoscopic remission (inactive group) and 145/181 (80%) had disease activity (active group) according to MES and SES-CD for UC and CD, respectively. On the other hand, 38/157 (24.2%) had a histologic remission, and 119/157 (75.7%) presented histological activity at the biopsy sampling at the index colonoscopy.

### Inflammatory biomarkers and indices

#### Endoscopic activity

We found a statistical difference in the FC (*P* = 0.001), albumin (*P* = 0.01), neutrophil (*P* = 0.04), and platelet count (*P* = 0.002) between the inactive and active groups of IBD patients.

Concerning the median value of the calculated inflammatory indices, the active group presented a high value of SIRI (*P* = 0.01), NLR (*P* = 0.02), PLR (*P* = 0.04), SII (*P* = 0.004), and AISI (*P* = 0.007) compared to the inactive group (Table 2).

**Table 2.** Baseline inflammatory biomarkers and indices concerning the endoscopic activity

Variable	Patients with inactive endoscopy median (range)	Patients with active endoscopy median (range)	*P* value*
N.	36/181 (20%)	145/181 (80%)	
Fecal calprotectin (mg/kg)	31.3 (3.8–402)	192 (0–3770)	0.001
C-reactive protein (mg/L)	1.4 (0.05–15.56)	1.69 (0–279)	0.28
Albumin (g/dL)	4.30 (3.72–4.97)	4.01 (2.45–4.88)	0.01
Lymphocytes (X10^3^/µL)	2 (0.73–4.56)	2.05 (0.5–23.7)	0.96
Neutrophil (×10^3^/uL)	4 (0.66–10.05)	4.63 (0.7–228)	0.04
Platelets (×10^3^/uL)	234.5 (134–506)	272 (0.7–651)	0.002
Monocytes (×10^3^/uL)	0.51 (0.21–0.90)	0.53 (0.08–5.3)	0.28
Eosinophils (×10^3^/uL)	0.15 (0–0.66)	0.13 (0–1.05)	0.89
SIRI	0.97 (0.20–4.47)	1.15 (0.15–20.1)	0.01
NLR	1.90 (0.31–7.48)	2.34 (0.43–49.6)	0.02
PLR	114.25 (46.9–348.9)	134.59 (0.14–833.3)	0.04
LMR	4.04 (1.66–11.7)	3.86 (0.31–24.7)	0.25
ELR	0.06 (0–0.23)	0.06 (0–0.7)	1.00
ENLR	0.28 (0–1.29)	0.29 (0–6.4)	0.38
CAR	0.45 (0.02–2.7)	0.42 (0–102.2)	0.54
SII	465.93(55.5–1971.6)	655.13 (8.9–8266.6)	0.004
MLR	0.25 (0.09–0.6)	0.26 (0.04–3.25)	0.25
AISI	216.66 (36.6–1237.2)	326.65 (9.07–10811.9)	0.007

AISI, aggregate index of systemic inflammation; AUC, area under the curve; CAR, C-reactive protein albumin ratio; CALPRO, calprotectin; CI, confidence interval; CRP, C-reactive protein; ELR, eosinophil-to-lymphocytes ratio; ENLR, eosinophil and neutrophil-to-lymphocytes ratio; LMR, lymphocytes-to-monocytes ratio; MLR, monocytes-to-lymphocytes ratio; NLR, neutrophil-to-lymphocytes ratio; PLR, platelets-to-lymphocytes ratio; SII, systemic immune inflammation index; SIRI: Systemic inflammation response index.

* *P* < 0.05 was considered statistically significant.

#### Histological activity

Concerning the measured biomarkers and the histological evaluation, we found a statistical difference for the FC (*P* = 0.04) and CRP (*P* = 0.01) only, among the inactive and active groups. For the calculated inflammatory indices, we found a significant difference for SIRI (*P* = 0.02), NLR (*P* = 0.02), SII (*P* = 0.02), and CAR (*P* = 0.02) between the inactive and active groups (Table 3).

**Table 3.** Baseline inflammatory biomarkers and indices concerning histologic activity

Variable	Patients with inactive histology Median (range)	Patients with active histology Median (range)	*P* value^a^
N.	38/157 (24%)	119/157 (76%)	
Fecal calprotectin (mg/kg)	50.00 (0–2167)	133.25 (5.4–3770)	0.04
C-reactive protein (mg/L)	1.00 (0.02–15.56)	2.07 (0–279)	0.01
Albumin (g/dL)	4.26 (3.1–4.9)	4.06 (2.45–4.8)	0.09
Lymphocytes (×10^3^/uL)	2.00 (0.7–3.7)	2.12 (0.5–23.7)	0.43
Neutrophil (×10^3^/uL)	3.82 (0.6–12.7)	4.70 (1.38–228)	0.007
Platelets (×10^3^/uL)	246.50 (174–529)	265.00 (0.7–651)	0.33
Monocytes (×10^3^ uL)	0.52 (0.28–1.07)	0.56 (0.08–5.30)	0.27
Eosinophils (×10^3^ uL)	0.12 (0–0.4)	0.14 (0–1.05)	0.73
SIRI	0.90 (0.2–6.12)	1.22 (0.19–20.13)	0.02
NLR	1.76 (0.3–11.7)	2.34 (0.6–49.6)	0.02
PLR	129.18 (59.8–427.7)	125.49 (0.14–833.3)	0.88
LMR	4.02 (1.66–9.05)	3.81 (0.31–24.7)	0.83
ELR	0.05 (0–0.24)	0.06 (0–0.74)	0.79
ENLR	0.22 (0–1.29)	0.33 (0–6.4)	0.08
CAR	0.18 (0–2.73)	0.54 (0–102.2)	0.02
SII	479.41 (55.5–5441.3)	596.57 (8.9–8266.6)	0.02
MLR	0.25 (0.11–0.6)	0.26 (0.4–3.25)	0.83
AISI	228.92 (36.6–2829.4)	326.65 (9.07–10811.93)	0.08

AISI, aggregate index of systemic inflammation; AUC, area under the curve; CAR, C-reactive protein albumin ratio; CALPRO, calprotectin; CI, confidence interval; CRP, C-reactive protein; ELR, eosinophil-to-lymphocytes ratio; ENLR, eosinophil and neutrophil-to-lymphocytes ratio; LMR, lymphocytes-to-monocytes ratio; MLR, monocytes-to-lymphocytes ratio; NLR, neutrophil-to-lymphocytes ratio; PLR, platelets-to-lymphocytes ratio; SII, systemic immune inflammation index; SIRI: Systemic inflammation response index.

a *P* < 0.05 was considered statistically significant.

### Receiver operating characteristics analysis and optimal cutoff of inflammatory indices

We conducted the ROC curve analysis for both the standard biomarkers (CRP and calprotectin) and all the inflammatory indices, to determine the test accuracy and specific cutoff values to identify endoscopic or histologic activity.

#### Endoscopic activity

**Fig. 1.**
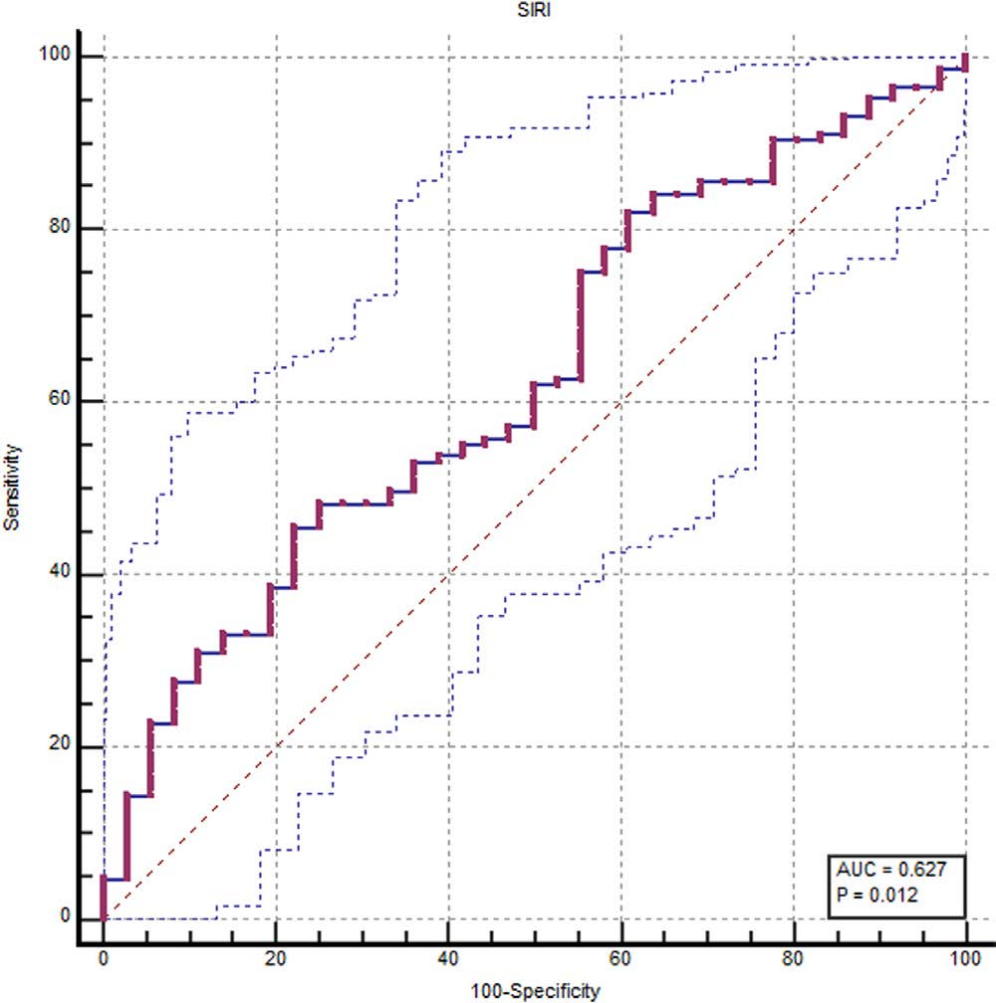
Receiver operating characteristics (ROC) analysis concerning SIRI and endoscopic activity. AUC, area under the curve; SIRI, systemic inflammation response index.

Table 4 reported the data concerning the test accuracy of the calculated inflammatory indices, CRP, and FC. At the ROC analysis, FC showed the best test accuracy (AUC, 0.765; CI, 0.642–0.863), as well as SIRI (AUC, 0.627; CI, 0.552–0.698) (Fig. 1). Moreover, the ROC analysis showed a suboptimal AUC for NLR and PLR (AUC, 0.620; CI, 0.545–0.691 and 0.607; CI, 0.532–0.679, respectively) (Table 4).

**Table 4.** Cutoff according to Youden Index for significant inflammatory indices for endoscopic and histological activity

Variable	Endoscopy activity					Histologic activity				
	Cutoff	Sen (%)	95% CI	Spe (%)	95% CI	Cutoff	Sen (%)	95% CI	Spe (%)	95% CI
CAR						>0.22	75.38	63.1–85.2	62.50	35.4–84.8
NLR	>2.32	51.03	42.6–59.4	75	57.8–87.9	>1.94	63.87	54.6–72.5	65.79	48.6–80.4
PLR	>147	42.76	34.6–51.2	80.56	64.0–91.8					
SIRI	>1.29	45.52	37.2–54.0	77.78	60.8–89.9	>0.75	80.67	72.4–87.3	39.47	24.0–56.6

CAR, C-reactive potein albumin ratio; CI: confidence interval; NLR, neutrophil-to-lymphocytes ratio; PLR, platelets-to-lymphocytes ratio; SEN, sensitivity; SIRI, systemic inflammation response index; SPE, specificity.

#### Histological activity

**Fig. 2.**
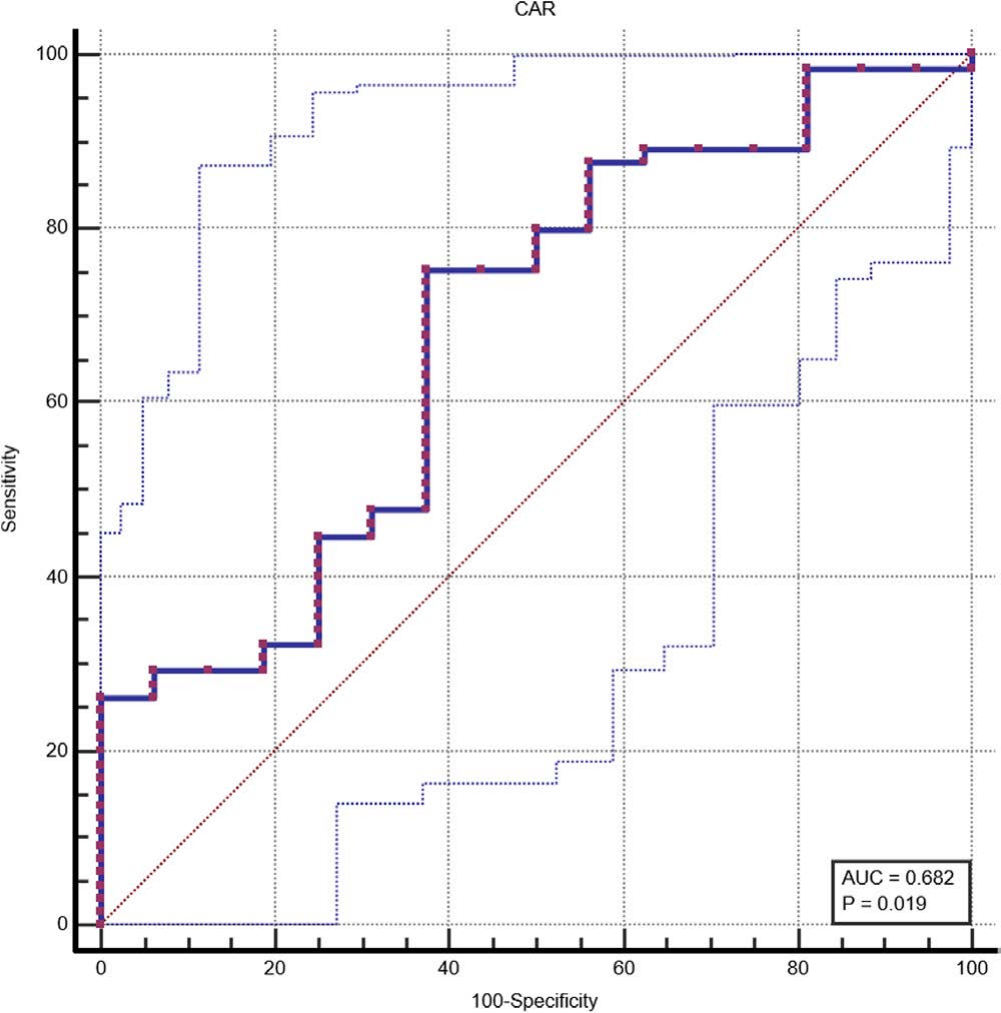
Receiver operating characteristics (ROC) analysis concerning CAR and histological activity. AUC, area under the curve; CAR, C-reactive protein albumin ratio

Concerning histological activity, the CAR presented the higher test accuracy among the calculated inflammatory markers (AUC, 0.682; CI, 0.569–0.781) (Fig. 2). FC and CRP also showed good test accuracy (AUC, 0.667; CI, 0.529–0.788 and AUC, 0.649; CI, 0.562–0.729, respectively) The SIRI and NLR presented a subdued diagnostic performance; the AUC was 0.622 (CI, 0.542–0.698) and 0.620 (CI, 0.544–0.701), respectively (Table 5).

**Table 5.** Receiver operating characteristics curve values of the inflammatory indices analyzed for endoscopic and histological activity

Variable	Endoscopy activity		Histologic activity	
	AUC	95% CI	AUC	95% CI
AISI	0.566	0.459–0.668	0.528	0.414–0.640
CAR	0.546	0.439–0.650	0.682	0.569–0.781
ELR	0.500	0.425–0.575	0.514	0.433–0.595
ENLR	0.547	0.472–0.621	0.594	0.513–0.672
LMR	0.562	0.487–0.636	0.511	0.431–0.592
MLR	0.556	0.449–0.659	0.511	0.431–0.592
NLR	0.620	0.545–0.691	0.625	0.544–0.701
PLR	0.607	0.532–0.679	0.508	0.427–0.588
SII	0.565	0.458–0.668	0.513	0.400–0.626
SIRI	0.627	0.552–0.698	0.622	0.542–0.698
CALPRO	0.765	0.642–0.863	0.667	0.529–0.788
CRP	0.561	0.480–0.641	0.649	0.562–0.729

AISI, aggregate index of systemic inflammation; AUC, area under the curve; CAR, C-reactive protein albumin ratio; CALPRO, calprotectin; CI, confidence interval; CRP, C-reactive protein; ELR, eosinophil-to-lymphocytes ratio; ENLR, eosinophil and neutrophil-to-lymphocytes ratio; LMR, lymphocytes-to-monocytes ratio; MLR, monocytes-to-lymphocytes ratio; NLR, neutrophil-to-lymphocytes ratio; PLR, platelets-to-lymphocytes ratio; SII, systemic immune inflammation index; SIRI: Systemic inflammation response index.

#### Optimal cutoff

Subsequently, for inflammatory indices that had shown acceptable AUC at ROC curve analysis (CAR, NLR, PLR, SIRI, FC, and CRP), suitable cutoffs were chosen to distinguish active and inactive patients, both endoscopically and histologically (Table 4).

According to Youden’s index test, the optimal cutoff for endoscopic activity was assessed as follows: for FC a value of 84 mg/kg (specificity 81.25% and sensitivity 70.2%); for CRP a value of 8 mg/L (specificity 93.75% and sensitivity 26.02%); for SIRI a value of 1.29 (specificity 77.78% and sensitivity 45.52%); for NLR a value of 2.32 (specificity 75.0% and sensitivity 51.03%), for PLR a value of 147.09 (specificity 38.89% and sensitivity 73.10%). For histologic activity prediction, these cutoffs were assessed: for FC a value of 64 mg/kg (specificity 65.0% and sensitivity 72.2%); for CRP a value of 1.1 mg/L (specificity 60.0% and sensitivity 65.71%); for SIRI a value of 0.75 (specificity 39.47% and sensitivity 80.67%); for NLR a value of 1.97 (specificity 65.79% and sensitivity 63.87%), for CAR a value of 0.22 (specificity 62.50% and sensitivity 75.38%).

## Discussion

The results reported in the present study have shown wide heterogeneity in terms of test accuracy of the evaluated inflammatory biomarkers and indices for IBD disease activity assessment. At the ROC analysis, the FC and SIRI presented the highest test accuracy for the endoscopic activity evaluation (AUC, 0.765; CI, 0.642–0.863; AUC, 0.627, CI, 0.552–0.698, respectively), while CAR, FC and CRP for histological activity (AUC, 0.682; CI, 0.569–0.781; AUC, 0.667; CI, 0.529–0.788; AUC, 0.649; CI, 0.562–0.729, respectively).

Few studies analyzed the role of these inflammatory scores in IBD. A recent retrospective study involving 187 UC patients explored the role of many integrated inflammatory indexes. Among others, they found that NLR and PLR were higher among active patients compared with patients in remission; moreover, NLR, PLR, CAR, and CRP-to-lymphocyte ratio (CLR) were higher in the cases of severe UC, and CLR had the greatest predictive accuracy for severe UC (AUC, 0.732). CAR and CLR resulted higher in 5-ASA nonresponder patients, with a greater predictive accuracy of CAR (AUC, 0.781) [38]. A retrospective study among 306 UC patients found that PLR and NLR were elevated in the active UC group compared with remission patients (defined by Truelove and Witts criteria). The authors defined the optimal cutoff for active UC 2.19 for NLR (sensitivity 78.8% and specificity 65%) and 147.96 for PLR (sensitivity 58.3% and specificity 75%) [39]. A multicentric study among 88 UC patients starting anti-TNF therapy, demonstrated both NLR and PLR as possible early predictors of therapeutic response [40]. A recent retrospective study including CD patients with a history of ileocolonic resection proposed a value of NLR > 2.45 as an independent risk factor for clinical recurrence [41].

A plethora of biomarkers for the IBD activity evaluation, with different pros and cons, already exist [8,42,43]. FC represents the most accurate test [8]. However, the collection mode and its cost are two non-negligible limits. The goal of our study arises from the need to have additional inflammatory indices that can help the clinician monitor disease activity, driving the daily clinical practice. The inflammatory indices evaluated in the present study are cheap and easily applicable compared to FC. These tests derive from simple mathematical formulas obtainable from a blood count with a leukocyte formula (Supplementary Table 1, Supplemental digital content 1, http://links.lww.com/EJGH/B60). Moreover, in some countries, the FC analysis represents an extra cost for the patients.

Thus, the useful application area for these inflammatory indices is represented by the identification of high-risk patients in terms of endoscopic or histological activity helping for a better selection of patients to undergo endoscopic examination.

A treat-to-target approach to identifying patients with disease activity (endoscopic or histological) despite clinical remission is an issue of extreme importance [44].

The more ambitious goal of endoscopic and histological remission leads to a lower risk of clinical relapse, and a higher quality of life in IBD patients [45,46]. Thus, having diagnostic tools cheaper and easier to use is a very relevant issue.

Anyway, we have calculated 10 of the most frequently applied and studied inflammatory indices in other diseases, but unfortunately, these inflammatory indices were found to be suboptimal for use in a practical clinical setting in everyday life, therefore we believe that research should concentrate its efforts on the research for new biomarkers that can really have implications in clinical practice, in the management, and therefore in the clinical course of IBD.

Although the reported results are enough robust, the inherent limits of retrospective design should be considered (missing data on endoscopic and concurrent biochemical evaluation have led to excluding a high number of patients). Moreover, patients who had an available endoscopic evaluation are more likely to have active disease, and for this reason, our cohort of patients is not fully representative of the outpatient reality.

On the other hand, we chose to evaluate the IBD target such as clinical activity, endoscopic activity, and histological activity in comparison to biochemical parameters, with very rigorous and reproducible criteria obtaining robust and reproducible results. Moreover, the evaluation of 10 inflammatory markers makes our study extremely complete concerning this field compared to others reported in the literature.

Among the evaluated inflammatory indices, the SIRI and the CAR presented the best test accuracy in an outpatient setting to identify endoscopic and histological activity despite clinical remission in IBD patients. However, globally the test accuracy of all the evaluated inflammatory indices appears to be suboptimal.

## Acknowledgements

All authors approved the final draft submitted. Each one of the authors was involved in the writing and revision of the manuscript.

C.N.: conception of the work, data collection, write the original draft; V.M.: methodological assessment, write the original draft; M.S.: data collection, write the original draft; S.G.: write the original draft and revision of the manuscript; F.S.: write the original draft; F.V.: revision of the manuscript; N.S.: statistical analysis; V.A.: interpretation of data and critical revision of the manuscript; L.G.: interpretation of data and critical revision of the manuscript.

### Conflicts of interests

There are no conflicts of interest.

## Supplementary Material


