# Supplementary material for: The role of new inflammatory indices in the prediction of endoscopic and histological activity in inflammatory bowel disease patients
Source: Eur J Gastroenterol Hepatol. 2024 Sep 12;37(1):24–32. doi: 10.1097/MEG.0000000000002842 (PMC11608588; doi:10.1097/MEG.0000000000002842)
Supplement: Supplementary file 1 [file ejgh-37-024-s001.pdf]

Supplementary Table 1 - Formulas to calculate the inflammatory indices.

INFLAMMATORY INDICES	FORMULAS
**Systemic inflammation response index (SIRI)**	(Neutrophil x Monocytes) / Lymphocytes
**Neutrophil-to-Lymphocytes ratio (NLR)**	Neutrophil / Lymphocytes
**Platelets-to-Lymphocytes ratio (PLR)**	Platelets / Lymphocytes
**Lymphocytes-to-Monocytes ratio (LMR)**	Lymphocytes / Monocytes
**Eosinophil-to-Lymphocytes ratio (ELR)**	Eosinophil / Lymphocytes
**Eosinophil and Neutrophil-to-Lymphocytes ratio (ENLR)**	(Eosinophil x Neutrophil) / Lymphocytes
**C-Reactive Protein Albumin ratio (CAR)**	C-Reactive Protein / Fecal Calprotectin
**Systemic immune inflammation index (SII)**	(Platelets x Neutrophil) / Lymphocytes
**Monocytes-to-Lymphocytes ratio (MLR)**	Monocytes / Lymphocytes
**Aggregate index of systemic inflammation (AISI)**	(Neutrophils × Platelets x Monocytes) / Lymphocytes
